# Urodynamic assessment of urinary incontinence

**DOI:** 10.4103/0970-1591.65392

**Published:** 2010

**Authors:** Sarah L. Housley, Chris Harding, Robert Pickard

**Affiliations:** Department of Urology, Freeman Hospital, Freeman Road, High Heaton, Newcastle upon Tyne, NE7 7DN, UK; 1 Newcastle University, Institute of Cellular Medicine, 3^rd^ Floor William Leech Building, The Medical School, Framlington Place, Newcastle upon Tyne, NE2 4HH, UK

**Keywords:** Review, urinary incontinence, urodynamics

## Abstract

**Context:**

Urodynamic evaluation in the assessment of women complaining of urinary incontinence remains controversial with recent UK National Institute of Health and Clinical Excellence guidance maintaining that it is unnecessary prior to surgery for women with a primarily stress leakage. Other experts contend it should be part of routine preoperative assessment since it establishes a diagnosis, allows more careful patient counseling and predicts surgical outcome.

**Objectives:**

To summarize current literature to define the evidence level on which these conflicting opinions are based.

**Materials and Methods:**

A systematic literature search was performed and retrieved publications summarized in a narrative evidence review using both original papers and previous reviews.

**Results:**

Five hundred and one primary research papers and 65 previous reviews were retrieved. The findings were summarized in a narrative comprising overview, description of methods of bladder and urethral pressure measurement, and a summary of the literature concerning four key questions.

**Conclusion:**

The level of evidence was low regarding answering each of the questions posed, preventing firm conclusions. Urodynamic findings do correlate with relevant symptoms and, to some extent, with symptom severity, giving reasonable diagnostic accuracy. There is no reliable evidence that preoperative urodynamic diagnosis improves outcome from surgery for stress incontinence although it is likely to facilitate preoperative discussion. Tests to differentiate sphincter deficiency and urethral hypermobility are not currently recommended due to poor validity and reproducibility. This along with the current use of mid-urethral tapes as the universal primary surgical procedure means differentiation is not a necessity. Preoperative diagnosis of detrusor overactivity does not appear to worsen surgical outcome in women with a primary symptom of stress leakage. Large, well-designed prospective studies are now underway to provide a definitive answer to these questions.

## INTRODUCTION

### Scope of review

Urinary incontinence has a significant impact on the quality of life of the affected individual and carries a substantial financial burden on any healthcare system. A shift to less invasive techniques has meant surgery is more accessible to patients and is being performed with increasing frequency. A key clinical research question in this area is where urodynamic diagnostics tests should be placed in the assessment pathway. This review highlights relevant findings from current literature to help guide clinicians regarding the use of cystometry in the investigation of urinary incontinence.

### Normal continence

The lower urinary tract which includes the bladder, urethra and sphincter mechanisms has two key functions - storage and voiding. Satisfactory storage requires intra-vesical pressure to remain lower than the pressure required to open the urethra. This is achieved by the elastic properties of the connective tissues of the bladder wall and the ability of the detrusor to increase its muscle fiber length which together ensure high compliance. Reflex sphincter activity guards against sudden intra-vesical pressure increases, such as those caused by standing and coughing, by maintaining urethral closure pressure higher than bladder pressure. When micturition is appropriate external urethral sphincter and pelvic floor relaxation initiates urine flow which is then augmented by detrusor contraction. Normal cystometry shows a low and stable bladder pressure during storage with no phasic activity and an appropriate increase in pressure during voluntary voiding.

### Incontinence definitions

In an effort to standardize terms and improve assessment, the International Continence Society (ICS) has published a number of standardization reports which define incontinence according to symptoms and findings on cystometry [Boxes 1 and 2].[1–4]

**Box 1:** ICS definition of symptoms

Urinary incontinence (UI) - “complaint of involuntary loss of urine”. Stress urinary incontinence (SUI) - “complaint of involuntary loss of urine on effort or exertion or on sneezing or coughing”. Urgency urinary incontinence (UUI) - “complaint of involuntary loss of urine accompanied or immediately preceded by urgency”. Nocturnal enuresis (NE) - “complaint of involuntary loss of urine which occurs during sleep”. Mixed urinary incontinence (MUI) - “complaint of involuntary loss of urine associated with urgency and also with exertion, effort or on sneezing or coughing”. Continuous urinary incontinence- “complaint of continuous leakage”.

**Box 2:** ICS definitions of urodynamic diagnoses

Urodynamic stress incontinence (UDSI) - “is noted during filling cystometry and is defined as the involuntary leakage of urine during increased abdominal pressure, in the absence of a detrusor contraction”[4] Detrusor overactivity incontinence (DOI) - “is incontinence due to an involuntary detrusor contraction”.[4]

Blaivas categorized urodynamic stress urinary incontinence into four types and subtypes according to findings on video cystometry [Table 1].[5]

Any situation where intra-vesical pressure is higher than urethral closure pressure will result in urinary leakage and this can be due to either decreases in urethra closure pressure caused by urethral dysfunction or increases in bladder pressure caused by detrusor dysfunction or a combination of both. Given this simple unifying urodynamic explanation it is not surprising that clinical tests measuring bladder or urethral pressure have been developed to delineate urodynamic diagnosis for women with urinary incontinence.

**Table 1:** Anatomical definitions of urodynamic stress incontinence according to Blaivas

Type	Urodynamic findings vesical neck and urethra		Cystocele
	Rest	Stress	
0	Closed, at superior symphysis pubis	Descend and open	No
I	Closed, at inferior symphysis pubis	Open and descend <2 cm	Little or none
II A	Closed, at inferior symphysis pubis	Open and descend >2 cm	Obvious cystocele
II B	Closed, below inferior symphysis pubis	May or may not descend but proximal urethra opens	Yes or no
III	Open in absence of detrusor contraction	Open in absence of detrusor contraction	Yes or no

### Aim of review

To critically review current literature concerning the usefulness of urodynamic testing for people with urinary incontinence in terms of diagnosis, communication and prognosis.

## MATERIALS AND METHODS

We carried out a systematic search of Medline using the MESH terms ‘urodynamics’ and ‘urinary’ and ‘incontinence’ between the years 1990 and 2009. We also searched references listed in existing reviews. The title and abstracts were checked by one author and full papers retrieved if relevant information regarding the use of urodynamics and incontinence assessment were found.

## FINDINGS

### Overview

The high number of papers retrieved highlights how broad this field is [Table 2]. An overview of this literature shows that the main purpose of urodynamics is considered to be as a tool to identify those women with urodynamic stress incontinence (diagnosis), to allow informed discussion concerning options of invasive treatment (communication) and to allow predication of outcome from surgery (prognosis). A COCHRANE review of this subject by Glazener and Lapitan[6] found 52 studies with patients randomized according to the performance of urodynamic tests. After quality scoring only three of these trials were eligible for meta-analysis.

**Table 2:** Numbers of publications retrieved by search strategy

Search criteria	Number of articles	Number of reviews
Urodynamics	1783	189
Urinary	200947	15948
Incontinence	26593	3639
Urodynamics and urinary and incontinence	501	65

### Description of the tests

#### Conventional cystometry

Filling and voiding cystometry is the most commonly performed procedure. It has a number of accepted and debated indications for use [Box 3].

**Box 3:** Indications for cystometry in people complaining of urinary incontinence

Accepted indications for cystometry
Uncertain diagnosis Clinical suspicion of mixed urinary incontinence Previous surgery for stress incontinence Symptoms suggestive of voiding dysfunction Incontinence associated with neurological disease
Debated indications for cystometry
Primary diagnosis of stress urinary incontinence Primary diagnosis of urgency incontinence (overactive bladder syndrome)

This test involved the placement of fluid-filled or solid-state pressure transducers into the bladder and rectum together with a fine catheter to fill the bladder artificially; allowing simultaneous measurement of intra-abdominal and intra-vesical pressure. Subtraction of these recorded pressures calculates the subtracted bladder pressure (detrusor pressure). While the bladder is filled artificially observations and measurements regarding detrusor overactivity, bladder capacity, bladder sensation, compliance and provoked or unprovoked leakage can be made. At the maximum cystometric capacity a voiding study is performed measuring the pressure and flow characteristics of micturition. Filling of the bladder with radio-opaque contrast and the use of fluoroscopic screening during filling, provocation and voiding is termed video urodynamics; this allows urinary leakage to be observed together with anatomical detail of the outlet during provocation and voiding. Provocation testing is ideally performed at a bladder volume > 150 ml using standardized increases in intra-abdominal pressure produced by coughing or valsalva maneuver to measure the pressure required to overcome urethral opening pressure; abdominal leak point pressure (ALPP) or valsalva leak point pressure (VLPP). Alternatively, provocation can be used to induce involuntary detrusor contractions such as turning on a water tap.[2]

#### Ambulatory cystometry

Ambulatory bladder monitoring is performed using the same principles as conventional cystometry but without any artificial filling; the patient’s bladder fills normally by their own urine production. This allows miniaturization of monitoring equipment leaving the patient free to move around. It is thought to be more representative of day-to-day life and may give more accurate information regarding lower urinary tract function. Usually, at least two filling and voiding cycles are captured over a four-hour period during which the patient drinks normally and keeps a diary of symptoms and events while a portable recording device continually measures intra-abdominal and intra-vesical pressure. The traces should be interpreted with the patient present to allow more diagnostic information to be collected. The catheter positions should be checked periodically.[7]

#### Urethral pressure profilometry – Dynamic and static

Urethral pressure profilometry is a technique designed to measure urethral closure pressure and is defined as the difference between the intra-luminal pressure in the urethra and the intra-vesical pressure at rest or during stress. For continence to be achieved, urethral pressure must exceed intra-vesical pressure at all times, except for micturition. In the static test a catheter with two separate transducers is placed with the top one in the bladder and the more distal one in the mid-urethra connected to a gradual infusion. In the dynamic test the catheter is withdrawn until the maximum urethral pressure is reached. Stress tests are used to illicit leakage.[8]

#### Noninvasive urethral pressure device

Urethral retro-resistance profile is a noninvasive technique involving the retrograde infusion of fluid against the closed sphincter allowing measurement of the pressure required to open the sphincter. Only a few studies have evaluated this technique but initial results were thought to be promising; differentiating women with incontinence from continent controls. There is also evidence it may be able to categorize patients into symptom severity but further studies are needed to replicate the findings of the originators of the technique.[9]

### Description of relevant studies

The ICS have detailed precise reports on urodynamic techniques in an effort to create standardized methodology; these should be consulted for more in-depth information.[2,10–12]

The goal of urodynamics is to reproduce the patient’s symptoms and provide a physical explanation of their cause; in this case to demonstrate incontinence and to differentiate between sphincter weakness – urodynamic stress incontinence, and involuntary bladder activity – detrusor overactivity incontinence (diagnosis). Various parameters are then used to try and distinguish between symptom severity, urethral hypermobility and intrinsic sphincter deficiency, and identify patients who have detrusor overactivity to facilitate discussion of treatment options (communication). The final aim is to be able to predict treatment outcome enabling the patient and clinician to choose the most appropriate intervention (prognosis).

#### Do urodynamics correlate with clinical history and symptom scores?

Lemack and Zimmern studied the predictive value of the Urogenital Distress Inventory (UDI-6) questionnaire for urodynamic outcomes in a retrospective study of 174 women. No single question was able to predict patients who had urodynamic stress incontinence or detrusor overactivity incontinence on cystometry. Combining a high score to the question related to leakage with physical activity and a history of previous incontinence surgery identifies 91% of ‘critical’ diagnoses in women with a predominant symptom of stress incontinence, urodynamic stress urinary incontinence with detrusor overactivity, abdominal leak point pressure < 60 cmH_2_O, or detrusor overactivity without urodynamic stress urinary incontinence in case of suspected stress urinary incontinence.[13]

Colli *et al*, performed a literature review including 5192 women with incontinence from 23 studies to identify how well incontinence symptoms related to urodynamic findings. The sensitivity was 0.82, 0.69 and 0.51 for stress, urgency and mixed urinary incontinence respectively. They therefore suggested that cystometry was more accurate for the diagnosis of women with stress incontinence. The specificity of urodynamics was similar in all the three groups.[14]

Another large retrospective study by Digesu *et al*, including 3428 women found that only 9% could be classified with stress urinary incontinence through the King’s Health Questionnaire.[15] Of this group, 78% were diagnosed as having urodynamic stress incontinence with the remainder having detrusor activity incontinence or inconclusive studies. The authors conclude that over 20% of women may not be best served by surgery as first-line treatment therefore justifying the routine use of urodynamics for primary diagnosis.

A small observational study by Fitzgerald and Brubaker[16] correlated symptoms of stress incontinence with urodynamic findings in 82 women. Of those women with severe symptoms, 89% had urodynamic stress incontinence. Severity did not correlate with diagnosis of intrinsic sphincter deficiency, and questions relating to urgency and frequency did not correlate with detrusor overactivity on cystometry.

Hashim and Abrams explored whether urgency was related to a urodynamic diagnosis of detrusor overactivity in a study of 1809 patients with overactive bladder syndrome.[17] They found that 69% of men and 44% of women with urgency had detrusor overactivity on cystometry, which increased to 90% of men and 58% of women if only those with urgency urinary incontinence were considered. Homma *et al*, using a small cohort of 30 patients showed that detrusor overactivity is not easily reproducible on repeat cystometry with 10% of patients having stable detrusor pressure on their second investigation; in addition 70-80% of patients had significant changes in other measured urodynamic parameters.[18]

All the evidence is conflicting; symptom scores do not appear to reliably predict urodynamic findings and are therefore not a replacement for cystometry in the assessment of patients with incontinence.

#### Recent guidelines state that cystometry is not required in ‘uncomplicated’ stress urinary incontinence cases – how valid is this recommendation?

Cystometry can characterize detrusor overactivity incontinence and urodynamic stress incontinence with an accuracy ranging from 60–90%, and the results help facilitate discussion of treatment options with the patient. It remains uncertain, however, how well a preoperative urodynamic diagnosis predicts outcome of treatment and consequently whether testing is cost-effective.

There are no large randomized trials comparing patients undergoing surgery for stress urinary incontinence with or without preoperative urodynamics, only small retrospective studies. One study of 212 women undergoing retropubic surgery for stress urinary incontinence compared three groups; Group One had basic assessment and video cystometry, Group Two basic assessment only, and Group Three had basic assessment and cystourethroscopy. Follow-up was over a 14-year period by annual questionnaire. No difference was found in postoperative continence rates between the three groups.[19]

Laurikainen and Kiiholma performed mid-urethral tape procedures (TVT) on 191 patients based on a clinical assessment alone and compared the outcome to historical cohorts of women who underwent preoperative cystometry. They found no difference in cure rates for incontinence (88% in both groups).[20]

It is clear that guidelines are based on small retrospective studies which did not show that urodynamics predicted outcome. To improve the level of evidence for this contention, large prospective randomized studies comparing standardized urodynamics with clinical assessment are currently being planned and conducted in the UK and United States.

#### Can urodynamics assess severity and distinguish between Intrinsic Sphincter Deficiency and urethral hypermobility?

Currently, the ICS does not recommend sub-categorization of urodynamic stress incontinence according to pressure or anatomical criteria such as Blaivas score, ALPP or VLPP.

A reliable and reproducible marker of severity of stress urinary incontinence has long been searched for with clinical measures such as questionnaires, pad tests and incontinent episodes, and urodynamic measures such as abdominal leak point pressurenone and urethral pressure profilometry all having been investigated.

It is well documented that women with stress urinary incontinence have much lower maximum urethral closure pressures than those without, with the magnitude of difference being dependent on symptom severity.[21,22] One study did find that a maximum urethral closure pressure < 20 cmH_2_O correlated with poor surgical outcome.[23] The wide overlap in pressure ranges between patients and normal controls and poor reproducibility has however limited the clinical usefulness of urethral pressure profile measurement.[24]

Abdominal leak point pressurenone measurements are reproducible but the lack of standardized methodology makes it difficult to compare studies. An early study by McGuire *et al*, demonstrated an inverse relationship between abdominal leak point pressure and severity of stress urinary incontinence.[25] Using a cohort of 125 women with stress urinary incontinence they were able to define a group with abdominal leak point pressure < 60 cmH_2_O and Blaivas Type Three urodynamic stress incontinence, a group with abdominal leak point pressure 60-89 cmH_2_O and Blaivas Type Two stress urinary incontinence and finally a group with abdominal leak point pressure > 90 cmH_2_O and Blaivas Type One incontinence. They suggested that the measurement could guide surgeons on which intervention to use; autologous bladder neck slings for women with values < 60 cmH_2_O (Type Three), for example. A later study examined 79 women who underwent both abdominal leak point pressure measurement and urethral pressure profilometry; all had urodynamic stress urinary incontinence without previous surgery and were planned to undergo insertion of mid-urethral tape (TVT).[26] Cutoff values of 60 cmH_2_O for abdominal leak point pressure and 30 cmH_2_O for maximum urethral closure pressure were used to categorize the women. No statistical association between abdominal leak point pressure and maximum urethral closure pressure was found; suggesting they are measuring different pathophysiological events. Lemack has subsequently published many papers concerning the diagnostic accuracy and clinical usefulness of both abdominal leak point pressure and urethral profile profilometry[27,28] in which he questions the validity of comparing data from different centers given variation in technique and comments on the conflicting data regarding their usefulness. In addition the clinical need to differentiate between intrinsic sphincter deficiency and urethral hypermobility on the basis of using different surgical techniques has been questioned. The previous practice of using bladder neck suspension for hypermobility and bladder neck slings for intrinsic sphincter deficiency has changed with most authorities recommending a mid-urethral tape as the primary procedure for all women with urodynamic stress incontinence and reserving the other techniques for secondary surgery. In support of this Bai *et al*, reported a study of 362 women who underwent mid-urethral tape insertion (TVT) which found no difference in outcome when stratified into intrinsic sphincter deficiency and urethral hypermobility groups according to abdominal leak point pressure and maximum urethral closure pressure measurements.[29] In a further study of 437 women undergoing mid-urethral tape insertion (TVT or TOT), the outcome for those undergoing TVT showed no association with abdominal leak point pressure or maximum urethral closure pressure measurements. In women undergoing TOT, however, those with measurements suggesting intrinsic sphincter deficiency had significantly worse outcomes.[30] Overall, at present it does not appear that differentiating between intrinsic sphincter deficiency and urethral hypermobility is of any clinical relevance prior to primary surgery.

#### Does a urodynamic diagnosis of detrusor overactivity affect outcome from stress urinary incontinence surgery?

Patients with stress urinary incontinence often have concurrent symptoms of urgency and frequency defining overactive bladder syndrome, and in a proportion this will be reflected by a finding of detrusor overactivity on cystometry. It is of some concern that the preoperative presence of detrusor overactivity will prejudice the outcome of surgery for presumed outlet weakness. Lai *et al*, have reviewed the literature on treatment of mixed urinary incontinence to gauge the impact that a prior diagnosis of detrusor overactivity had on treatment outcome.[31] They quoted a number of uncontrolled studies to have shown that in approximately 50% of cases the symptoms of overactive bladder resolve after bladder outlet surgery for stress incontinence.[32–35] Lai *et al*. conclude that patients with mixed urinary incontinence can be treated with colposuspension and tape procedures. If urgency symptoms persist they can be treated with conventional treatment modalities. A further insight was made by an additional study of 35 women with mixed urinary incontinence which found that those with persistent detrusor overactivity after surgery also had a low maximum flow rate preoperatively.[36] These small studies indicate the complex etiology of mixed urinary incontinence which is yet to be fully understood.

## CONCLUSION

Although significant research regarding clinical and urodynamic assessment of urinary incontinence exists, an extensive and authoritative review by Martin *et al*, commissioned by the UK National Health Service found few well-powered primary studies, making valid conclusions difficult.[37] A thorough history and examination are always essential and, together with simple clinical measurements such as urinalysis, pad tests, fluid charts and post-void residual, will normally enable clinicians to select non-surgical interventions such as drugs or pelvic floor muscle training without need for invasive urodynamics.

The degree to which cystometry affects surgical decision-making and prediction of outcomes is unclear. The evidence was of low level; consisting mainly of small case series. This, together with a lack of standardization in urodynamic terms and techniques in the literature makes meaningful interpretation and comparison of studies difficult. The limited evidence available does point to a degree of correlation between symptom severity, urodynamic diagnosis and surgical outcome. The validity and clinical usefulness of urethral closure pressure assessment remains subject to much discussion due to poor reproducibility, uncertain ability to differentiate between intrinsic sphincter deficiency and urethral hypermobility, and lack of influence of deciding on the primary surgical procedure. There is therefore a widely acknowledged need for large, well-designed prospective studies to address these issues and these are now in progress.
